# Management of Food-Related Diarrhea Outbreak in the Emergency Department: Lessons Learned from the German STEC O104:H4 Epidemic

**DOI:** 10.1155/2015/480680

**Published:** 2015-10-11

**Authors:** Friedhelm Sayk, Niels Henrik Asselborn, Nora Eisemann, Alexander Katalinic, Jörg Metzner, Sebastian Wolfrum, Klaus Fellermann, Johannes Knobloch, Martin Nitschke

**Affiliations:** ^1^Department of Internal Medicine II/Emergency Department, University Hospital of Schleswig-Holstein, Campus Lübeck, 23560 Lübeck, Germany; ^2^Department of Internal Medicine I, University Hospital of Schleswig-Holstein, Campus Lübeck, 23560 Lübeck, Germany; ^3^Institute for Clinical Epidemiology, University of Lübeck, 23560 Lübeck, Germany; ^4^Emergency Department, Sana-Kliniken, 23560 Lübeck, Germany; ^5^Institute of Medical Microbiology and Hygiene, University Hospital of Schleswig-Holstein, Campus Lübeck, 23560 Lübeck, Germany

## Abstract

Emergency department (ED) management of the German STEC O104:H4 outbreak in 2011 was not limited to patients being truly infected with STEC. In parallel to spread of alarming news in public media, patients suffering from diarrhea due to other reasons fearfully presented, equally. We retrospectively characterized these two cohorts for anamnestic, clinical, and laboratory findings at their first ED contact. From 15th of May to July 2011, 302 adult patients with diarrheal complaint presented at the EDs of two tertiary hospitals in Lubeck, northern Germany. Fecal testing for STEC was obtained in 245 (81%) patients: 105 were STEC-positive and 140 were STEC-negative. Anamnestic characteristics (defecation rate, visible bloody diarrhea, and lower abdominal pain), abdominal tenderness, and some laboratory findings were significantly different between both cohorts but not reliable to exclude STEC. In >90% of STEC-positive patients diarrheal symptoms had started in May, reflecting the retrospective nationwide peak of infections, whereas the majority of STEC-negative patients became symptomatic in June 2011. During the German STEC O104:H4 outbreak a definite distinction at initial ED contact between STEC-positive versus STEC-negative patients by clinical judgment alone was not reliable. Fecal testing in the ED, however, might survey the outbreak of foodborne infections with the utmost precision.

## 1. Introduction

**Figure 1:**
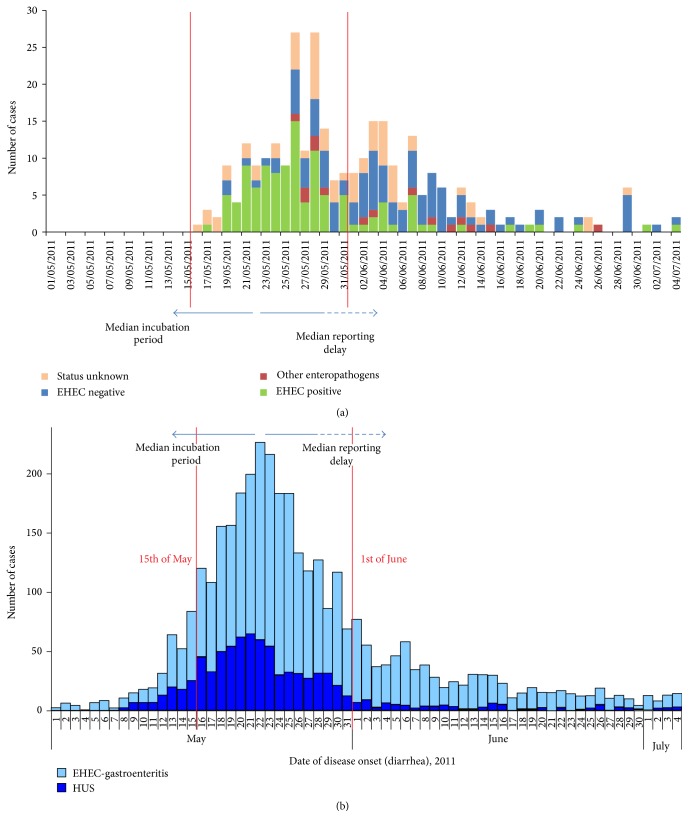
(a) Date and number of patients presenting at the ED in Lubeck with suspected EHEC-infection. The group was subdivided into patients with approved STEC-infection, patients with stool enteropathogens other than STEC, and patients without any fecal pathogens and those who have no valid STEC-result. (b) Nation-wide epidemiology of diarrhea onset in patients with approved STEC-infection with or without hemolytic uremic syndrome (HUS). Peak of diarrhea onset was at 21st of May; arrow to the left side indicates median incubation period of eight days and arrow to the right side indicates median reporting delay of > one week (adapted from [1]).

In 2011, a large outbreak of Shiga toxin-producing* Escherichia coli* (STEC O104:H4) has caused 3816 documented infections in Germany, including 845 confirmed cases of hemolytic uremic syndrome (HUS). According to retrospective epidemiologic analyses of the Robert-Koch Institute (RKI), which is the leading national German health authority, about 90% of diarrheal cases occurred during the second half of May (Figure 1(b)) [1]. During the outbreak period, however, this clear-cut epidemiologic frame was unknown. The first official statement about the outbreak was given on May 20th [2], and an official declaration that the epidemic had ended was published on 26th of July. Likewise disease communication in public media (TV news, press coverage, etc.) continued until July 2011 including alarming reports that reinforced uneasiness in the population.

Emergency departments (EDs) at tertiary hospitals offer service 24 h/7 d. Therefore EDs are prone to a multitude of first medical contacts during outbreaks with foodborne diarrheal pathogens. A rapidly acting network between EDs and health authorities might significantly improve infection surveillance and accelerate outbreak control. In the context of STEC-outbreaks the special challenge for the ED is to detect and manage (a) STEC-infected patients presenting with variable clinical manifestations, (b) to distinguish patients suffering from diarrhea due to other infectious or noninfectious reasons, and (c) to screen ED presenters driven by anxiety rather than objective clinical findings. This resource-demanding aspect is neglected in epidemiologic reports and most scientific work-up of the German STEC/HUS-outbreak 2011, though it was of high, yet undetermined social and health-economic impact.

During the outbreak a definite distinction at initial ED contact between STEC-infected and noninfected patients by pure clinical judgment was not possible and clinical criteria indicating risk of future HUS-development were unknown. Therefore, stratification for the need of follow-up was not possible until microbiological results of fecal testing were available. Here we report anamnestic, clinical, and laboratory characteristics of STEC-positive compared to STEC-negative outbreak-related ED contacts from May to July 2011 in Luebeck, one of the most affected cities in northern Germany. Almost all patients were subject to standardized anamnestic, clinical, and laboratory assessment and fecal microbiologic testing was initiated. Comparing both cohorts retrospectively we reflect the impact of EDs for infection surveillance and outbreak control.

## 2. Patients and Methods

During the outbreak period all adult patients were documented who presented to any one of two tertiary hospital EDs in Luebeck, the university hospital (UKSH) and the municipal hospital (Sana-Klinik), with any complaint consistent with STEC-infection including diarrheal symptoms, visible blood in stool, “bleeding hemorrhoids,” or personal fear of being infected. The first official press release about the outbreak was given by the RKI on May 20th [2]. Starting from 21st of May history taking was standardized with a questionnaire that was filled in by the patients and/or the ED doctors. This included the following questions: beginning of diarrheal symptoms, estimated number of (bloody) stools within last 24 h, upper/lower/diffuse abdominal pain, fever, nausea and vomiting, alimentary details, and history of travel.

Stool specimen sampling was rigorously performed since 20th of May 2011 using standard methods for* E. coli* culture and Shiga toxin detection. The outbreak strain was characterized as an extended-spectrum *β*-lactamase- (ESBL-) expressing* E. coli* of serotype O104:H4 with virulence factors of both enterohemorrhagic* E. coli* (EHEC) and enteroaggregative* E. coli* (EAggEC) [3]. Starting from 23rd of May stool culture for the outbreak strain was performed by screening for ESBL expressing* E. coli* (confirmed by VITEK 2 and *E*-Test, bioMérieux, Marcy l'Etoile, France) on culture media selective for STEC-serotypes attaining a high sensitivity. This was combined with testing for the presence of a Shiga toxin encoding phage confirmed by phenotypic Shiga toxin expression (as detected by RIDASCREEN Verotoxin ELISA, R-Biopharm AG, Darmstadt, Germany) effectuating high specificity. Additionally, in patients with proven STEC-infection serotype O104:H4 was confirmed by PCR as suggested by the Robert Koch Institute [4]. Using this diagnostic strategy, STEC O104:H4 infection was confirmed or excluded with very high sensitivity and specificity.

Five patients who had presented with respective symptoms between May 15th and 20th were contacted by hospital staff at the earliest possibility in order to obtain fecal testing and to establish follow-up for the rule-out of HUS-development. The survey was continued until July 2011, the time when German health authorities officially declared that the outbreak time period had stopped.

According to our ED standard-operating procedure, vital signs and body temperature were measured in almost every outbreak-related presenter. Moreover abdominal tenderness and bowel sounds were examined clinically and routine laboratory testing including HUS-indicating parameters was performed. The decision for hospital admission versus outpatient management was made according to the ED physician's clinical judgment. Outpatients who did not provide fecal specimen during their ED visit received a prepaid small package to send a stool sample at earliest possibility. All outbreak-related ED presenters who were not admitted received written instruction to establish clinical and laboratory follow-up at their general practitioner to rule out HUS development.

At the beginning of August 2011, all outbreak-related ED-presenters were contacted by postal mail and asked to fill in a supplemental questionnaire which served for cross-checking and/or completing anamnestic and anthropometric data. The survey and questionnaire were approved by the local ethics committee as “ad hoc” decision on 25th of May.

## 3. Statistics

Patients were retrospectively divided into two cohorts, STEC-positive and STEC-negative, according to their fecal microbiology result. STEC-negative patients were subdivided into those with fecal results positive for common enteropathogenic bacteria (other than STEC) or viruses and those with completely negative stool specimen.

We characterized these cohorts for anamnestic, clinical, and laboratory findings at their first ED contact and compared STEC-positive and STEC-negative (total and subgroups: “without stool pathogens” and “other stool pathogens”) patients using Student's *t*-tests and *χ* ^2^-tests. Notably, due to the ESBL-selective testing mentioned above the sensitivity for the detection of other bacterial pathogens was limited, and, therefore, the definition of the subgroups of STEC-negative patients may be not completely reliable. A multiple logistic regression analysis was conducted to estimate odds ratios for positive versus negative STEC-infection. Anthropometric, anamnestic, clinical, and laboratory parameters were selected based on univariate testing and on clinical knowledge and were entered into the regression model.

## 4. Results

**Figure 2:**
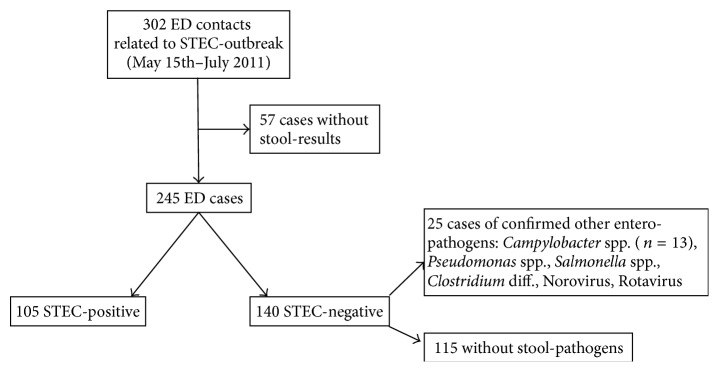
Enrollment of outbreak-related ED patients into the analysis.

During the STEC O104:H4 outbreak period, 302 adult patients with diarrheal complaint presented at the ED of any one of two tertiary hospitals in Luebeck. Fecal specimen was obtained in 245 (81%) patients: 105 were STEC-positive and 140 were STEC-negative; 25 patients of the STEC-negative cohort were positively tested for common enteropathogens (other than STEC). 57 (19%) did not return stool samples and were excluded from further analysis (Figure 2).

As shown in Table 1 anthropometric characteristics did not differ between both cohorts. Anamnestically, STEC-positive patients had a higher number of stools within last 24 h and had stronger complaint of lower abdominal pain. Visible bloody diarrhea was reported by 86% of STEC-positive compared to 59% of STEC-negative patients. Vital signs including body temperature, however, did not differ. At clinical examination 66% of STEC-positive versus 41% of STEC-negative patients had tenderness on abdominal palpation. At laboratory analysis the STEC-positive cohort showed slightly higher neutrophil counts and higher LDH, serum-creatinine, and bilirubin levels. These laboratory differences were consistent even if those cases were excluded who were already diagnosed for manifest HUS at their initial ED contact (*n* = 17).

**Figure 3:**
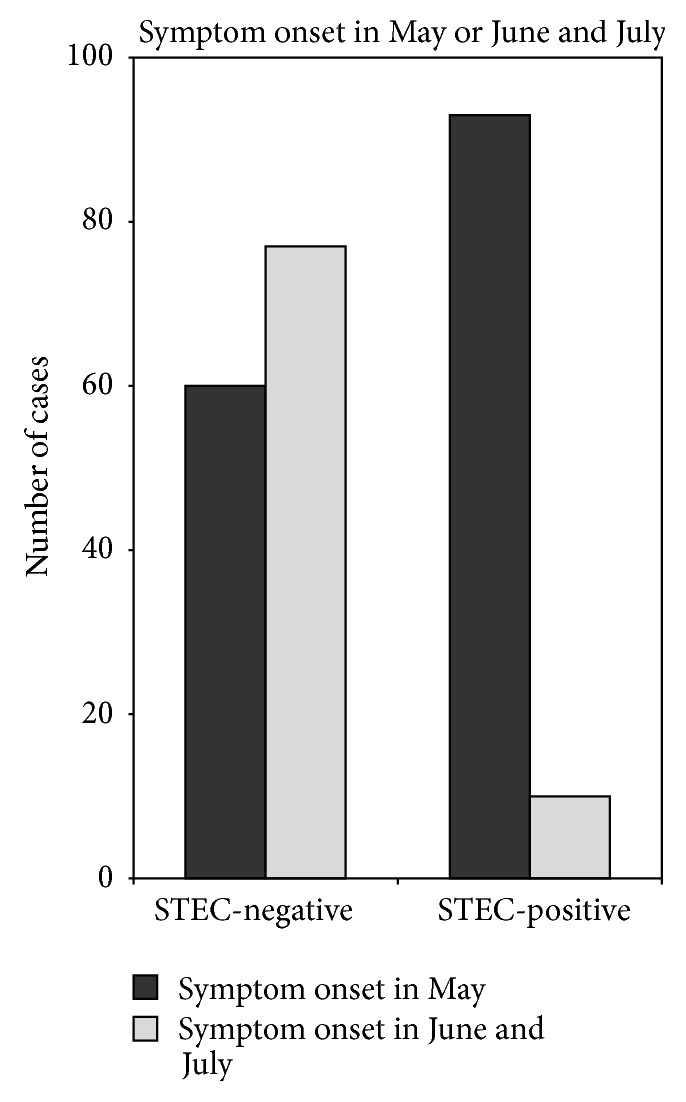
Month of onset of diarrheal symptoms in STEC-positive and STEC-negative ED contacts.

However, the most striking difference between both groups was the time of symptom onset: in >90% of STEC-positive patients diarrheal symptoms had started in May 2011, whereas about 55% of STEC-negative presenters reported that diarrheal symptoms had started in June (Figure 3). As depicted in Figure 1(a) the daily numbers of ED contacts showed two overlapping clusters: the first occurred during the second half of May and was dominated by STEC-positive patients; the second phase occurred during the first half of June and was characterized by STEC-negative presenters.

## 5. Logistic Regression Analysis

Data consistency of most parameters was >80–90% except for BMI and defecation rate. However, due to missing items the multivariate logistic regression analysis was limited to 143 of 245 patients. We found that visible bloody diarrhea independently increased the risk of STEC-disease about 16-fold. The clinical finding of lower abdominal tenderness increased the risk about 5-fold. A defecation rate > 10/24 h did not attain statistical significance due to those 25 STEC-negative patients who were positive for other enteropathogens. Elevated blood-leukocyte counts were associated with an increased risk of STEC-infection. However, the parameter that was most significantly linked to STEC-positivity was the time period of diarrhea onset: the beginning of gastroenterocolitic symptoms in May 2011 increased the risk of STEC-infection about 40-fold (Table 2).

## 6. Discussion

The main finding of the present observational study is that the ED management of the German STEC O104:H4 outbreak involved not only STEC-positive patients, but at even greater quantity STEC-negative ED contacts. This phenomenon of “STEC + X” is largely ignored by epidemiologic statistics. As depicted in Figure 1(a) the daily numbers of outbreak-related ED contacts were not uniformly distributed. Roughly two main overlapping clusters could be distinguished: The first peaked during the second half of May with the majority of patients tested positively for STEC-infection. This early cluster closely corresponds to the Gaussian distribution of the nationwide epidemiology curve that was presented by the RKI in September 2011 (Figure 1(b)) [1]. Slight differences between the nationwide curve (Figure 1(b)) with peak on May 22nd and our local data (Figure 1(a)) with peak on May 26th might result from the fact that date of ED contact (local Figure 1(a)) includes some delay from start of diarrheal symptoms (nationwide Figure 1(b)).

The second cluster of ED presentations peaked at about 4th of June. This second wave of contacts is dominated by STEC-negative subjects. Interestingly, the days of highest contact rates do well coincide with alarming regional news in public media: on May 28th STEC-related deaths including one old patient in Luebeck were reported [5]; on June 4th a well-known local restaurant was identified for dissemination of STEC-contaminated food [6]. The seriousness of the illness and the fatalities, coupled with the lack of a definitive source of the causative agent, created uneasiness among the public. Obviously, the local involvement into the STEC-tragedy further boosted ED contact rates.

The phenomenon that the media coverage might significantly increase public uneasiness with alarming reports is well-known from the H1N1-pandemics [7]. While it is the business of public media to sell news, wild headlines are not helpful to gaining public trust and cooperation with health agencies in controlling the spread of an outbreak. Moreover, during the German STEC-O104:H4 outbreak, a close epidemiologic surveillance was significantly delayed due to bureaucratic decentralized reporting pathways involving local, federal state, and national levels of health authorities [8]. The mean reporting delay was estimated 1-2 weeks [1]. Given the fact that the outbreak strain showed a prolonged median incubation period of 8 days up to the onset of diarrheal symptoms, as compared to experience from previous outbreak investigations with EHEC O157 (3 to 4 days), public and official awareness of the epidemic was congruent with the second peak of ED contacts (mainly STEC-negative) but not with the real peak of the infection interval (Figure 1(b)).

Since bloody diarrhea is frequently the first symptom that EHEC patients experience, the development of an EHEC outbreak can be assessed almost real-time by ascertaining patients presenting with these symptoms, for example, in emergency departments [9]. Therefore, on May 27, 2011, syndromic surveillance of patients with bloody diarrhea was established in collaboration of emergency departments and the RKI [8]. This still was subject to some bias but effectively helped to correct the official estimate of the outbreak during June 2011 [1].

At initial ED contact a definite distinction of STEC-positive and STEC-negative patients by clinical judgment alone was not possible and clinical criteria for the risk of future HUS-development were unknown. In the light of our early clinical experience since May 15th that even oligosymptomatic patients with only mild STEC-gastroenteritis might deteriorate to severe HUS within few days, stratification for the need of follow-up was not possible until microbiological results of the respective fecal testing were available. According to the “Practice Guidelines for the Management of Infectious Diarrhea” and recent recommendations [10, 11] gastroenteritic symptoms with passage of small-volume stool containing visible blood as well as a suspected outbreak should prompt fecal culture of enteropathogenic bacteria including STEC. Therefore, stool sampling was rigorously initiated either during the ED or hospital stay or via packages with prepared sampling kits. Though some of these kits were not returned, we have valid STEC-results in >80% of ED presenters due to the very high sensitivity and specificity of the combined approach using ESBL-culture, ELISA, and PCR-tools.

The univariate and multivariate comparison of the outbreak-related STEC-positive versus STEC-negative ED cohort revealed that the visible presence of blood in the feces as well as lower abdominal tenderness was clearly associated to STEC-positivity. This is in accordance with previous reports [10]. A high defecation rate (>10/24 h) did not distinguish between STEC-infected cases and those patients who had positive fecal testing for enteropathogens other than STEC. This heterogeneous group of 25 patients, comprising 13 subjects infected with* Campylobacter* spp., was too small for an extended separate analysis. Though blood leukocyte counts, serum creatinine, and bilirubin showed statistic differences between both cohorts they did not reliably approve or exclude STEC-infection. Likewise other anamnestic, clinical, and laboratory parameters were not helpful for the discrimination between STEC-positive and STEC-negative cohorts. In contrast, the time of symptom onset was by far the strongest risk factor for STEC-infection. In our retrospective analysis >90% of STEC-positive patients developed diarrhea in May 2011.

## 7. Strength and Limitations

Inherent to the unpredictable nature of outbreaks this is an unplanned observational study. Hence, in our cohorts data are not complete for every patient who met the inclusion criteria. However, data consistency was >80–90% for all parameters except for BMI and defecation rate. Standardization of our anamnestic, clinical, and laboratory diagnostic and management early in the course of the outbreak provided sufficient statistical power. We did not aim at calculating a combined clinical risk score to estimate high probability versus exclusion of STEC-infection, because this approach appears to be of minor utility with regard to the overwhelming outbreak-related temporal frame.

## 8. Conclusion and Perspective

During the STEC O104:H4 outbreak in 2011 EDs had to manage STEC-positive as well as STEC-negative diarrhea patients. Though some anamnestic, clinical, and laboratory findings were significantly different between STEC-positive and STEC-negative cohorts, these parameters seem not suitable to reliably discriminate between both patient groups. The risk of STEC-positivity was tightly linked to the period of symptom onset corresponding to the peak of new infections in retrospective nationwide epidemiologic reports.

EDs offer contact to patients 24 h/7 days. They are strongly involved in the management of any infectious epidemic. Close collaboration with the microbiology department is mandatory for efficient surveillance. Bloody diarrhea is a well-established trigger of fecal diagnostic [9–11]. Subtype-specific microbiologic work-up for foodborne infections like STEC can identify whether sudden increases in reported cases are due to sporadic cases or to one or more outbreaks [12]. The costs and potential benefits of subtype-specific surveillance tools have been discussed elsewhere previously [13, 14]. Given the possibility of long-term-shedding in a high proportion of infected individuals, as found in the O104:H4 epidemic, such surveillance would be reasonable even beyond the mere outbreak period [15]. EDs are the optimal partners for central health authorities: close communication via direct reporting systems can avoid reporting delays and seismographically survey the outbreak. Good public communication is essential, but communication failures delay outbreak control, undermine public trust, and unnecessarily prolong economic, social, and political turmoil.

## Figures and Tables

**Table 1:** Anamnestic, clinical, and laboratory findings in STEC-positive versus STEC-negative ED patients. STEC-negative cohort was subdivided into patients without microbiological finding of fecal pathogens and those having enteropathogens other than STEC.

	STEC-positive (*n* = 105)	STEC-negative					
		Total (*n* = 140)	*p*	Without stool pathogen (*n* = 115)	*p*	Other stool pathogens (*n* = 25)	*p*
Anthropometric and anamnestic parameter							

Age (years, mean ± SD)	52.4 ± 20.2	43.1 ± 19.1	**<0.001**	43.5 ± 19.3	**0.001**	41.3 ± 18.4	0.614
BMI (kg/m², mean ± SD)	25.5 ± 5.5	25.5 ± 4.5	0.976	25.0 ± 4.4	0.531	27.6 ± 4.8	**0.028**
Female (% of patients)	57.1	63.6	0.308	64.3	0.274	60.0	0.682
Smoker (% of patients)	23.0	32.5	0.140	33.7	0.113	27.3	0.563
Start of symptoms (% of patients)							
May 2011	90.3	43.2	**<0.001**	41.7	**<0.001**	50.0	0.643
June/July 2011	9.7	55.4		56.2		50.0	
Defecation rate > 10/d (% of pat.)	56.9	23.2	**<0.001**	17.6	**<0.001**	52.9	**0.005**
Faeces “bloody” (% of patients)	86.4	58.7	**<0.001**	63.2	**<0.001**	37.5	**0.020**
Nausea (% of patients)	50.0	55.0	0.451	51.4	0.839	70.8	0.085
Vomiting (% of patients)	42.2	34.6	0.232	28.6	**0.037**	62.5	**0.002**
Abdominal pain (% of patients)	92.0	83.6	0.057	80.9	**0.020**	95.8	0.074
Pain at lower abdomen (% of patients)	71.0	41.0	**<0.001**	40.1	**<0.001**	41.7	0.283
Fever (>38°C; % of patients)	4.0	9.4	0.113	7.0	0.347	20.8	**0.035**

Clinical findings							

Body temperature (°C, mean ± SD)	37.1 ± 0.5	37.1 ± 0.7	0.682	37.0 ± 0.7	0.210	37.4 ± 0.7	**0.012**
Blood pressure (mmHg, mean ± SD)							
Systolic	133 ± 20	131 ± 22	0.468	132 ± 23	0.617	129 ± 20	0.554
Diastolic	78 ± 11	76 ± 11	0.141	77 ± 12	0.318	74 ± 9	0.202
Heart rate (min^−1^, mean ± SD)	80.4 ± 16.2	81.1 ± 15.8	0.758	79.9 ± 15.7	0.824	87.5 ± 15.2	0.060
Abdominal tenderness (% of patients)	65.6	40.9	**<0.001**	39.1	**<0.001**	50.0	0.342
Tenderness lower abdomen (% of patients)	56.3	26.2	**<0.001**	21.7	**<0.001**	36.4	0.651
Bowel sounds present (% of patients)	96.9	98.4	0.474	90.4	0.169	90.9	**0.003**
Signs of dehydration (% of patients)	31.9	18.0	**0.016**	12.3	**0.001**	45.5	**<0.001**

Laboratory findings							

Total leukocytes (/*µ*L, mean ± SD)	10725 ± 4286	9467 ± 3737	**0.019**	9230 ± 3712	**0.009**	10431 ± 3754	0.151
Creatinine (*µ*mol/L, mean ± SD)	116.7 ± 150.1	71.3 ± 18.7	**0.001**	70.7 ± 16.5	**0.002**	73.8 ± 26.2	0.464
LDH (U/L, mean ± SD)	270.3 ± 334.2	172.3 ± 42.4	**0.001**	170.5 ± 41.7	**0.003**	179.5 ± 45.6	0.354
Total bilirubin (*µ*mol/L, mean ± SD)	18.2 ± 25.7	10.4 ± 7.7	**0.002**	9.4 ± 5.1	**0.001**	14.9 ± 13.4	**0.002**
CRP (mg/L, mean ± SD)	25.6 ± 33.4	27.3 ± 52.7	0.775	15.4 ± 32.2	**0.032**	74.8 ± 84.4	**<0.001**
Thrombocytes (/nL, mean ± SD)	207 ± 79.0	221 ± 65.8	0.130	227 ± 53.5	**0.040**	197 ± 64.3	**0.018**
Hemoglobin (g/L, mean ± SD)	135 ± 24.9	137 ± 19.8	0.446	136 ± 19.9	0.686	141 ± 19.2	0.260
Hematocrit (/L/L, mean ± SD)	0.40 ± 0.1	0.41 ± 0.1	0.352	0.40 ± 0.0	0.567	0.42 ± 0.1	0.233

**Table 2:** Multivariate logistic regression analysis for STEC-positive versus STEC-negative ED contacts. Odds ratio indicates the respective risk of being STEC-infected.

	Odds Ratio	95% Confidence interval	*p* value
Start of symptoms (May versus June/July)	**40.885**	**(7.870–212.393)**	**<0.001**
Defecation rate (>10 versus <10/24 h)	1,993	(0.545–7.279)	0.297
Bloody diarrhea	**15.056**	**(2.014–112.558)**	**0.008**
Anamnestic lower abdominal pain	2.969	(0.824–10.673)	0.096
Lower abdominal tenderness	**4.637**	**(1.186–18.124)**	**0.027**
Elevated C-reactive protein	0.592	(0.137–2.562)	0.483
LDH (U/L)	1.011	(0.999–1.024)	0.082
Bilirubin (*µ*mol/L)	1.064	(0.981–1.155)	0.133
Creatinine (*µ*mol/L)	1.033	(1.001–1.065)	0.044
Elevated leukocyte count	**16.617**	**(2.316–119.243)**	**0.005**
